# Treatment of Uremia in Children by Pilocarpin

**Published:** 1882-07

**Authors:** 


### TREATMENT OF UREMIA IN CHILDREN BY PILOCARPIN.

From the study of eleven cases, all treated by muriate of pilocarpin, Dr. Praetorius, of Mayence, arrives at the following conclusions: The action of the alkaloid of jaborandi on children may be recognized by active carotid pulsation, reddening of the face, and profuse perspiration, which begins on the forehead, upper lip, and chin, and gradually extends over the whole body. These symptoms appear about three to five minutes after hypodermic administration of the drug. Accompanying the diaphoresis a profuse salivary secretion is observable. In infants the sialagogue action is the more reliable of the two. The temperature is affected only in so far as the evaporation from the sweating cutaneous surface produces a slight secondary lowering. The single dose of the drug is $\frac{1}{32}$ to $\frac{1}{3}$ of a grain (0.002–0.02 Gm.). The children, as a rule, complain of severe nausea, and vomiting is frequent. Conditions of slight collapse are sometimes noticeable.

The following *résume* of inferences is appended to the paper:

1. The treatment of uremia by hypodermic use of pilocarpin gives satisfactory results. It appears advisable to resort to this plan of treatment as soon as headache, an irregular pulse, and vomiting point to the probability of renal complications.

2. The contra-indications for its employment are, the presence of grave complications, abnormal weakness, collapse, or general cutaneous dropsy.

3. It appears that in "glomerular" nephritis pilocarpin fails to produce a beneficial effect; but as this variety of Bright's disease cannot be differentiated from other forms by our present method of examination, this condition cannot of course be classed with the contra-indications.

4. In addition to the diaphoretic action of the muriate of pilocarpin, a direct influence on the renal secretions appears to exist.—*Jahrb. fur Kinderheilkunde; Lond. Pract.*

